# Supplementary material for: Non-invasive assessment of steatohepatitis indicates increased risk of coronary artery disease
Source: PLoS One. 2023 Sep 28;18(9):e0286882. doi: 10.1371/journal.pone.0286882 (PMC10538770; doi:10.1371/journal.pone.0286882)
Supplement: S1 Table — (DOCX) [file pone.0286882.s001.docx]

**S1 Table.** Severity of liver disease on the subgroup of patients with MAFLD

| **Variables** | | **MAFLD cohort** | | | **Coronary Intervention** | | | | |
| --- | --- | --- | --- | --- | --- | --- | --- | --- | --- |
|  |  |  | |  | **Yes** | | **No** | |  |
|  |  |  | **n=49** | |  | **n=22** |  | **n=27** | **p values** |
| **Steatosis risk** |  |  |  | |  |  |  |  |  |
| **CAP (dB/m)** |  | 336 | (318;353) | | 346 | (326.5;353.8) | 332 | (331.5;350.5) | 0.20 |
| **S2/3** | **CAP>331** | 27 | 55,1% | | 13 | 59,1% | 14 | 51,9% | 0.61 |
| **Fibrosis risk** |  |  |  | |  |  |  |  |  |
| **Liver stiffness (kPa)** | | 4.9 | (4.1;5.9) | | 5.4 | (4.4;6.2) | 4.6 | (3.6;5.9) | 0.19 |
| **Low risk** | **<8kPa** | 44 | 89,8% | | 19 | 86,4% | 25 | 92,6% | 0.47 |
| **Increased risk** | **≥8kPa** | 5 | 10,2% | | 3 | 13,6% | 2 | 7,4% |  |
|  |  |  |  | |  |  |  |  |  |
| **NFS^a^ (n=47)** |  | -1.05 | (-1.77;-0.10) | | -1.1 | (-1.80;-0.49) | -0.78 | (-1.74;0.33) | 0.34 |
|  | **≥Sens. Cut-off** | 18 | 38,3% | | 5 | 25,0% | 13 | 48,1% | 0.11 |
|  | **≥Spec. Cut-off** | 6 | 12,8% | | 2 | 10,0% | 4 | 14,8% | 0.63 |
| **FIB4^b^ (n=48)** |  | 1.46 | (1.07;1.85) | | 1.55 | (1.37;2.02) | 1.35 | (1.02;1.75) | 0.22 |
|  | **≥Sens. Cut-off** | 21 | 43,8% | | 11 | 52,4% | 10 | 37,0% | 0.29 |
|  | **≥Spec. Cut-off** | 3 | 6,3% | | 2 | 9,5% | 1 | 3,7% | 0.41 |
| **NASH risk** |  |  |  | |  |  |  |  |  |
| **AST % of ULN** | | 59 | (50.6;68.3) | | 63 | (51.8;84.4) | 58 | (50.6;66.3) | 0.18 |
|  | **AST > ULN** | 3 | 6% | | 3 | 14% | 0 | 0% |  |
| **FAST^c^** |  | 0.22 | (0.14;0.31) | | 0.26 | (0.22;0.40) | 0.18 | (0.13;0.25) | **0.012** |
|  | **≥Sens. Cut-off** | 11 | 22,4% | | 7 | 31,8% | 4 | 14,8% | 0.16 |
|  | **≥Spec. Cut-off** | 3 | 6,1% | | 3 | 13,6% | 0 |  |  |

Values given in median (IQR) and absolute numbers, %

MAFLD, metabolic associated fatty liver disease; CAP, Controlled Attenuation Parameter; NFS, NAFLD-Fibrosis Score; FIB4, FIB4-index; AST, aspartate aminotransferase; ULN, upper limit of normal; FAST, Fibrosis-AST-score

^a^ sensitive/specific cut-offs were −1.455 (age 36–65) and 0.12 (age ≥ 65)/0.676 (age ≥ 36)

^b^ sensitive/specific cut-offs were 1.3 (age < 65) and 2.0 (age ≥ 65)/2.67 (all ages)

^c^ sensitive/specific cut-offs were 0.35/0.67
