# Supplementary material for: Non-invasive assessment of steatohepatitis indicates increased risk of coronary artery disease
Source: PLoS One. 2023 Sep 28;18(9):e0286882. doi: 10.1371/journal.pone.0286882 (PMC10538770; doi:10.1371/journal.pone.0286882)
Supplement: S2 Table — (DOCX) [file pone.0286882.s002.docx]

**S2 Table.** Characterization of patients at high and low risk of relevant liver fibrosis

| **Variables** |  |  | **LSM <8kPa** |  | **LSM >8kPa** |  | **p-value** |
| --- | --- | --- | --- | --- | --- | --- | --- |
|  |  |  |  |  |  |  |  |
|  |  |  | **n=114** |  | **n=6** |  |  |
| **Age, years** |  |  | 65.4 | (58.3;73.8) | 64.4 | (57.9;66.1) | 0.45 |
| **Gender, male/female** | |  | 74/40 |  | 6/0 |  |  |
| **BMI, kg/m^2^** |  |  | 27.1 | (25.0;30.4) | 34.3 | (30.3;39.4) | **0.003** |
| **BMI >25 kg/m^2^** | |  | 84 | 73.7% | 6 | 100% |  |
| **High blood pressure** | |  | 81 | 71.1% | 5 | 83.3% | 0.52 |
| **Diabetes mellitus** | |  | 27 | 23.7% | 3 | 50% | 0.15 |
| **Relevant alcohol consumption** | | | 46 | 40.4% | 4 | 66.7% | 0.20 |
| **Waist-hip ratio** | |  | 0.97 | (0.9;1.0) | 1.02 | (1.0;1.1) | **0.023** |
| **CAD** |  |  | 47 | 41.2% | 3 | 50% | 0.67 |
| **CAP** |  |  | 284 | (244;326) | 345 | (324;362) | **0.008** |
| **MAFLD** |  |  | 44 | 38.6% | 5 | 83.3% | **0.030** |
| **NFS^a^ (n=111)** |  |  | -1.40 | (-2.22;-0.33) | -0.69 | (-1.65;0.35) | 0.25 |
|  | ≥Sens. Cut-off |  | 33 | 29.7% | 2 | 33.3% | 0.85 |
|  | ≥Spec. Cut-off |  | 8 | 7.2% | 1 | 16.7% | 0.40 |
| **FIB4^b^ (n=112)** | |  | 1.53 | (1.11;1.93) | 1.39 | (1.33;1.87) | 0.87 |
|  | ≥Sens. Cut-off |  | 50 | 44.6% | 3 | 50% | 0.80 |
|  | ≥Spec. Cut-off |  | 10 | 8.9% | 0 |  |  |
| **FAST^c^ Score** |  |  | 0.14 | (0.1;0.24) | 0.54 | (0.40;0.66) | **<0.001** |
|  | ≥Sens. Cut-off |  | 11 | 9.6% | 5 | 83.3% | **<0.001** |
|  | ≥Spec. Cut-off |  | 1 | 0.9% | 2 | 33.3% | **<0.001** |

Values given in median (IQR) and absolute numbers, %

LSM liver stiffness measurement; BMI body-mass index; CAD coronary artery disease; CAP, Controlled Attenuation Parameter; MAFLD, metabolic associated fatty liver disease; NFS, NAFLD-Fibrosis Score; FIB4, FIB4-index; FAST, Fibrosis-AST-score

^a^ sensitive/specific cut-offs were −1.455 (age 36–65) and 0.12 (age ≥ 65)/0.676 (age ≥ 36)

^b^ sensitive/specific cut-offs were 1.3 (age < 65) and 2.0 (age ≥ 65)/2.67 (all ages)

^c^ sensitive/specific cut-offs were 0.35/0.67
