# Supplementary material for: Non-invasive assessment of steatohepatitis indicates increased risk of coronary artery disease
Source: PLoS One. 2023 Sep 28;18(9):e0286882. doi: 10.1371/journal.pone.0286882 (PMC10538770; doi:10.1371/journal.pone.0286882)
Supplement: Attachment — Submitted filename: Response to reviewers.docx [file pone.0286882.s004.docx]

Dear editor, dear reviewers,

We thank you for your helpful comments and improved our manuscript accordingly. We hope to answer your comments and concerns sufficiently

For the ease of the reader our comments are written in blue color.

Yours sincerely,

Sebastian Beer, MD

Thomas Karlas, MD

On behalf of all co-authors

**EDITORS' COMMENTS TO THE AUTHOR:**

*1. Please ensure that your manuscript meets PLOS ONE's style requirements, including those for file naming. The PLOS ONE style templates can be found at 
https://journals.plos.org/plosone/s/file?id=wjVg/PLOSOne_formatting_sample_main_body.pdf and 
https://journals.plos.org/plosone/s/file?id=ba62/PLOSOne_formatting_sample_title_authors_affiliations.pdf*

We thank the editor for this opportunity. We fully revised the manuscript according to all style requirements of PLOSone including the file naming. We hope we did everything fully sufficiently.

*2. "Thank you for stating the following in the Competing Interests section: 
    "I have read the journal's policy and the authors of this manuscript have the following competing interests: TK and JW received an unrestricted research grant from Echosens, France. TK was an invited speaker for Echosens.SB, JB, MN and VB declared that no competing interests exist"*

*Please confirm that this does not alter your adherence to all PLOS ONE policies on sharing data and materials, by including the following statement: "This does not alter our adherence to PLOS ONE policies on sharing data and materials.” (as detailed online in our guide for authors http://journals.plos.org/plosone/s/competing-interests).  If there are restrictions on sharing of data and/or materials, please state these. Please note that we cannot proceed with consideration of your article until this information has been declared.*

*Please include your updated Competing Interests statement in your cover letter; we will change the online submission form on your behalf.*

We updated the statement for competing interests accordingly.

*3. PLOS requires an ORCID iD for the corresponding author in Editorial Manager on papers submitted after December 6th, 2016. Please ensure that you have an ORCID iD and that it is validated in Editorial Manager. To do this, go to ‘Update my Information’ (in the upper left-hand corner of the main menu), and click on the Fetch/Validate link next to the ORCID field. This will take you to the ORCID site and allow you to create a new iD or authenticate a pre-existing iD in Editorial Manager. Please see the following video for instructions on linking an ORCID iD to your Editorial Manager account:* [*https://www.youtube.com/watch?v=_xcclfuvtxQ*](https://www.youtube.com/watch?v=_xcclfuvtxQ)

We ensured that the ORCID iD of the corresponding author validated and updated.

*4. Please include captions for your Supporting Information files at the end of your manuscript, and update any in-text citations to match accordingly. Please see our Supporting Information guidelines for more information:* [*http://journals.plos.org/plosone/s/supporting-information*](http://journals.plos.org/plosone/s/supporting-information)*.*

We listed the Supporting Information accordingly at the end of the manuscript and updated the in-text citation.

*Additional Editor Comments:
Our reviewers found some merits in this article but made a number of comments and suggestions. I ask the authors to clarify/correct each of the points that the reviewers indicated below. Additionally, in the text and tables, the authors should report p values with up to 2 significant digits and maximum 3 decimals (p = 0.21, p = 0.007, p = 0.001, etc.).*

The p values have been adapted.

**PPOINT-TO-POINT RESPONSE TO REVIEWER'S COMMENTS TO THE AUTHOR**

**REVIEWER #1**

*Have the authors made all data underlying the findings in their manuscript fully available?*

We thank the reviewer for the comment. We updated the supporting file labeling and listed the files at the end of the manuscript. The complete raw data used for this manuscript is included in S1 Dataset.

*The authors have prospectively evaluated non-invasive fibrosis tests (FIB4, NFS, FAST and Fibroscan) in 120 patients with coronary arterial disease. MAFLD and advanced liver fibrosis (LSM ≥8 kPa) were observed in 41% and 6% (5% in the abstract?), respectively. A significant high score was present in patients needing coronary intervention.*

We thank the reviewer for note and corrected the discrepancy in the percentage.

*Paragraph “Introduction”

• Page 3, line 73-74: The authors could add the more recent reference by Toh MR, (Global epidemiology and genetics of hepatocellular carcinoma. Gastroenterology 2023 Feb 2:S0016-5085(23)00105-1.) Tho et al. indicated that “Non-cirrhotic HCC is more common in NASH than the other etiologies; 39% of NASH-associated HCCs develop in absence of cirrhosis, compared with 22%, 6%, 9% for HBV, HCV, ALD respectively.”*

We are thankful for the advice in additional references and amended accordingly.

*• Page 3, line 78: Please, indicate the precise prevalence with its CI : 44.6% (95% CI, 36.0%–53.6%). Interestingly, the prevalence of CAD was even more important when invasive coronary angiogram was used: 60.5% (95% CI, 43.8%–75.1%)*

We provided that important additional information fittingly.

*Paragraph “Materials and Methods”

• Page 4, line 128: The authors need to specify conditions of blood samples. Surely, in a fasting condition*

The missing statement “in an overnight fasting state” was added.

*• Page 5: The authors could add the reference by Castera L, et al (Gastroenterology 2019) for the threshold (8 kPa) used to define significant fibrosis*

The additional reference was included.

*• Page 5, line 147 : “Patients with SLCD exceeding 25 mm were examined with the XL-probe”. This recommendation is similar to that also proposed by EASL using a BMI cut-off of 30 kg/m2 to select the M or XL probe (EASL-ALEH clinical practice guidelines. J Hepatol 2015;63:237–264). The authors could add this BMI cut-off for clinicians since BMI cut-off is more often used than SLCD.*

We thank the reviewer for this suggestion. For routine care BMI might also be used to determine the choice of M vs. XL probe. However, we think that the choice according to SLD is more accurate. The recent version of the Fibroscan device is equipped with an automated-probe selection tool that is based on SLD assessment using the A-mode. Therefore, we used a standardized probe choice approach that has been described in Blank et al. (Dig Liver Dis. 2022 Mar;54(3):358-364. doi: 10.1016/j.dld.2021.08.003.) This paper also discusses the issue of a BMI based probe choice in more detail. We have added a reference to this paper.

*• Page 5, line152: The authors used AAP value ≥302 dB/m to diagnose any grade of steatosis. However, different cut-off values have been proposed elsewhere and, for EASL, a CAP (> 275 dB/m) is currently suggested (J Hepatol. 2021; 75:659-689). This point could be discussed.*

Recent analysis on the accuracy of CAP show that the specificity of this technology can be impaired by BMI and patients’ anthropometry, especially in NAFLD patients [Petroff, Blank et al. Lancet Gastroenterol Hepatol. 2021 Mar;6(3):185-198. doi: 10.1016/S2468-1253(20)30357-5.]. Therefore, the choice of the cut-off depends on the clinical strategy (“ruling in” vs. “ruling out”) and the local prevalence. The applied cut-off derives from the largest prospective trial on NAFLD screening using VCTE/CAP including > 400 biopsy-controlled data sets from 7 centers in the UK [Eddowes et al. Gastroenterology. 2019 May;156(6):1717-1730. doi: 10.1053/j.gastro.2019.01.042]. The screening approach and patients’ characteristics of our study has many parallels to that of Eddowes at al. Therefore, we refrain from introducing other cut-off values.

Therefore, we added in the discussion:

“Although there is no general recommended cut-off for CAP, we used the cut-offs proposed by Eddowes et al. [Ref], one of the largest prospective biopsy-controlled studies providing data in CAP accuracy for steatosis quantification.”

*Paragraph “Results”
• In this section, baseline biological and demographic characteristics of the 120 patients should be clearly reported in a dedicated table. All the components of the MAFLD definition need to be reported (waist circumference, hs-CRP, etc.) together with liver enzymes, PT, albumin, platelets, etc. and % of patients with MAFLD.*

We modified Table 1 accordingly by extending the reported values.

*• Instead of describing characteristics of patients with and without coronary intervention in the total cohort, the authors should complete this table 1 by describing the non-MAFLD population*

Modification of table 1 included a comparison of MAFLD and non-MAFLD patients.

*• In this section, the authors could specify the indications of routine (n= …) or emergency angiography (n=…).*

Number and indication of angiography were specified: “Of these 120 patients, n=19 (15.8%) underwent emergency CA due to (suspected) myocardial infarction. Indication for routine CA (n=101) was stable angina pectoris.”

*• Page 9 : the authors write “LSM was valid and reliable in 120 (94% of eligible) patients”. The 128 “eligible” patients could be shown in the flow chart (Figure 1) for a more comprehensive reading.*

Figure 1 was adjusted correspondingly.

*• Table 2 : the authors need to define the ULN of AST and ALT in their centre. All percentages have been rounded in this table. I suggest to indicate the right numbers (for instance 4/70 = 5.7% instead of 6%). The authors may round figures in the discussion*

The ULN have been added to the laboratory methods: “The upper limit of normal of AST at time of the study was 0.6 µkat/l in women and 0.85 µkat/l in men, and of ALT 0.58 µkat/l in women and 0.85 µkat/l in men.” The format of numbers was adapted.

*• Page 11, line 241 : the authors write “None of the high-risk FIB4 patients and only one of the high-risk NFS patients had increased LSM values”. I suggest to create a table in supplementary appendix to characterize the 113 patients with a low risk (< 8kPa) and the 7 patients with a high risk (≥ 8 kPa), even though the number of high-risk cases is low. We need to better characterize the small group of CAD patients with LSM ≥ 8 kPa*

We presented an additional S2 Table for a more detailed characterization of the patients with significantly elevated LSM and added to the results: “The small number of patients with significant elevated LSM ≥8kPa had significantly higher BMI, waist-to-hip ratio and CAP resulting in an increased prevalence of MAFLD and elevated FAST score (S2 Table).”

*• Table 3 : the title of this table does not indicate clearly what the authors are really looking for. I suggest to write “Variables associated with coronary intervention using multivariate analysis”.*

We modified the title according to the reviewer’s recommendation.

*• Page 11, line 249 : The authors write “The results are very similar in the MAFLD subgroup”. The authors provide in Suppl. Appendix regarding this multivariate analysis regarding the subgroup of 49 MAFLD patients. Since only 22 coronary interventions were performed in this subgroup, only two variables can be entered in the model.*

We thank the reviewer for this critical remark. We have adjusted the respective phrase accordingly: “In the MAFLD subgroup the values were similar although the limited patient number did not allow a multivariate logistic regression.”

*Paragraph “Discussion”

• Page 12, line 273-74: It is surprising that metabolic factors are not associated with advanced liver fibrosis in this study. Indeed, liver fibrosis development in NAFLD patients may result from a long-term exposure to cardiometabolic risk factors such as diabetes, arterial hypertension, dyslipidaemia and overweight (Baratta F, et al. Gastroenterol Hepatol 2020;18:2324-31). Genetic factors may also impact the relationship between NAFLD and CAD (Miao Z, et al. HGG Adv 2021 Aug 24;3:100056) ; The pathophysiology of the relationship between NAFLD and CAD could be explained briefly with the main causal factors.*

We thank the reviewer for this comment. We added the following in the discussion: “This might result from the relatively small study population and may also reflect the small effect size in preselected cases.” Further pathophysiological factors such as genetic alterations are involved in the relationship between NAFLD and CAD [(Miao Z, et al. HGG Adv 2021 Aug 24;3:100056] but could not be addressed in our study.

*• Page 12, line 285: “As a secondary finding, we observed associations of higher liver stiffness within the limits of normal in patients with relevant CAD compared to those without need of revascularization therapy”: these data are not shown.*

We endorsed our statement by calculation and the following additional statement: “Even after exclusion of the patients with LSM ≥8 kPa, difference remained significant (4.8 kPa vs. 4.1 kPa; p=0.018)”

*• Please, check the references 7, 8, 9, etc.*

The missing details have been added.

**Reviewer #2:**

*The study presents the results of an interesting and original research. Experiments, statistics, and other analyses are performed to a high technical standard and are described in a detailed manner.*

*Conclusions are presented in an appropriate fashion and are supported by the data, but the authors must describe if are any group of patients with coronary interventions that must do the non-invasive assessment of liver fibrosis, probably patients with metabolic syndrome or T2DM should be examined with transient elastography for liver fibrosis.*

We thank the reviewer for this critical remark and have adjusted the discussion accordingly:

“However, patients at risk for advanced liver disease (e.g. with metabolic syndrome) should undergo screening as recommended by current guidelines independent of the presence CAD. [Tacke F, et al.. Updated S2k Clinical Practice Guidelines: NAFLD. Z Gastroenterol. 2022; 60(9):e733-e801.; Visseren FLJ, et al. 2021 ESC Guidelines on cardiovascular disease prevention in clinical practice. Eur Heart J. 2021; 42(34):3227–337.]”

*Also, the authors must explain if are any correlations between LSM values and severity of CAD according to the grade of stenosis. Also if are any correlations between them in which patients are most commen met?*

We thank the reviewer for this suggestion. We had discussed this idea during the preparation of the study protocol, but finally chose to define a dichotomous endpoint (CAD present or not), because any further correlation with disease severity would go beyond the scope of a pilot study of this scale. Especially, the severity of CAD cannot be simply correlated with a single factor (such as the grade of stenosis), but would require more special methods including pressure analysis for estimation of coronary flow reserve and microvascular resistance index (Knuuti J, et al.: 2019 ESC Guidelines for the diagnosis and management of chronic coronary syndromes: The Task Force for the diagnosis and management of chronic coronary syndromes of the European Society of Cardiology (ESC). European Heart Journal 2020; 41 (3): 407–7).

We added a phrase to the discussion.

*In addition, they must put a space before the references in the text.*

This was corrected.
