# Supplementary material for: Non-invasive assessment of steatohepatitis indicates increased risk of coronary artery disease
Source: PLoS One. 2023 Sep 28;18(9):e0286882. doi: 10.1371/journal.pone.0286882 (PMC10538770; doi:10.1371/journal.pone.0286882)
Supplement: Attachment — Submitted filename: Response to the reviewers 2.docx [file pone.0286882.s005.docx]

Dear editor, dear reviewers,

We thank you for your comments and advices. We made all the changes according to the reviewer’s recommendation.

For the ease of the reader our comments are written in blue color.

Yours sincerely,

Sebastian Beer, MD

Thomas Karlas, MD

On behalf of all co-authors

**PPOINT-TO-POINT RESPONSE TO REVIEWER'S COMMENTS TO THE AUTHOR**

**REVIEWER #1**

Page 4, line 115-117: the references should be put at the end of the sentence: “….severe congestive heart failure …………… and pulmonary hypertension (WHO III or IV) (28, 29).”.

We corrected this accordingly.

Revised Table 1 : Quantitative data should be expressed in median with IQR and qualitative data in absolute numbers with %
For instance :
“Male gender n (%): 80 (67%)” and only one column is sufficient
The line “AST % of ULN” is write twice ?
AST should be expressed in µkat/L, like GGT and AP
Please correct “gGT” by “GGT”
Instead of “exp9/l”, write “109/L” or “mm-3”
Is is really useful to write “HbA1c NSGP/DCCT”? It should be simpler to write only “HbA1c”

The recommendations have been implemented. Formatting changes have been adapted also to table 2.

Concerning AST and ALT values: We use sex-specific cut-off values for these parameters. Therefore, the absolute values (in µkat/L) do not provide sufficient information when presented alone. That is why we initially decided to show %-values in relation to the upper limit of normal (ULN) and provided an additional line for categorial numbers, i.e. cases with increased ALT or AST, respectively. We now added absolute AST and ALT values to Table 1, but we abstained to do so in Table 2 for ease of reading.

Page 9, line 238: write “≥ S2”

We corrected accordingly.

Page 11 : In the following sentence, the authors could specify that “high-risk”patients were defined according to the specific cut-off of NFS : “Only 5 patients (10%) of the interventional group and 4 patients (6%) of the non- intervention group (p=0.36) were identified as high-risk”

We added to the sentence: “according to the specific cut-off of NFS”

Page 11, line 266: it would be better to write in full “MI” for a better understanding of this sentence

We made the change accordingly.

Reviewer #2: The work of Beer et al. is an exceptional original paper, which includes new data regarding liver fibrosis and CAD. Also, this paper open a new path in the links between liver fibrosis and CAD.

Thank you.
